# Multi-Omics Approaches for Revealing the Epigenetic Regulation of Histone H3.1 during Spermatogonial Stem Cell Differentiation In Vitro

**DOI:** 10.3390/ijms24043314

**Published:** 2023-02-07

**Authors:** Li Liu, Haojie Li, Mengjie Wang, Xiangzheng Zhang, Jie Ren, Yan Yuan, Jiahao Sha, Xuejiang Guo

**Affiliations:** 1 State Key Laboratory of Reproductive Medicine, Nanjing Medical University, Nanjing 210029, China; 2 State Key Laboratory of Reproductive Medicine, Women’s Hospital of Nanjing Medical University, Nanjing Maternity and Child Health Care Hospital, Nanjing Medical University, Nanjing 210029, China

**Keywords:** histone modification, spermatogonial stem cell, differentiation, transcription factor, epigenetic regulation

## Abstract

Epigenetic regulation, particularly post-translational modifications (PTMs) of histones, participates in spermatogonial stem cell (SSCs) differentiation. However, there is a lack of systemic studies of histone PTM regulation during the differentiation of SSCs due to its low number in vivo. Herein, we quantified dynamic changes of 46 different PTMs on histone H3.1 by targeted quantitative proteomics using mass spectrometry during SSCs differentiation in vitro, in combination with our RNA-seq data. We identified seven histone H3.1 modifications to be differentially regulated. In addition, we selected H3K9me2 and H3S10ph for subsequent biotinylated peptide pull-down experiments and identified 38 H3K9me2-binding proteins and 42 H3S10ph-binding proteins, which contain several transcription factors, such as GTF2E2 and SUPT5H, which appear to be crucial for epigenetic regulation of SSC differentiation.

## 1. Introduction

Spermatogonial stem cells (SSC) reside in the testes of male mammals, where they serve as progenitor cells for spermatogenesis. Following puberty, SSCs experience multiple physiological regulations, including meiosis, which promote their subsequent differentiation [1]. The continual SSC proliferation and differentiation constitute the foundation of spermatogenesis, ensuring the continued viability of male fertility. The SSC-induced meiosis is inextricably linked to both genetic and epigenetic co-regulations [2]. In the early 2000s, the successful establishment of a long-term SSC culture in vitro provided a solid platform for SSC modulation. Subsequently, the establishment of a robust and complete meiosis in vitro model that meets the “Gold Standard” of meiosis enabled investigation into the regulatory mechanism of SSC differentiation in vitro [3].

During the SSC differentiation process, epigenetic events induce dramatic alterations, namely, DNA methylation (DM), histone modification (HM), chromatin remodeling, RNA modifications, and so on. Many DM regulators, such as *Dnmt3a*, *Dnmt1*, and *Tet1*, are confirmed as modulators of SSC differentiation [4]. In addition to DM, HMs, particularly H3.1 modifications, are critical for SSC differentiation. However, the existing studies are mostly limited to single HMs, such as H3K4me3 and H3K27me3 [5,6]. There is currently a lack of systematic research on HM function in this process. Thus, it is imperative to systematically reveal the dynamic HMs alterations that occur during SSC differentiation in order to resolve this urgent issue.

Spermatogenesis is a continuous and asynchronous process regulated by a myriad of somatic cells, such as Sertoli and Leydig cells [7]. In a previous study, the researchers only elucidated the differentiation process of spermatogonia from the transcription level [8]. The high-purity isolation of all cell types from within the testis is key to studying spermatogenesis. In a prior investigation, researchers utilized WIN18446 to synchronize SSCs for spermatogenesis. In doing so, they revealed an essential role of FBXO47 during meiosis, thereby uncovering extensive dynamic processes and molecular signatures that modulate gene expression and germ cell development [9]. However, SSCs in vivo are rare as it is challenging to use these cells in vivo for proteomics research, particularly those involving histone post-translational modification (PTM). Given these challenges, it is essential to obtain massive quantities of cells of a single cell type to resolve this issue.

In this study, for the first time, we employed an in vitro model of SSCs differentiation to systemically identify the post-translational HM sites during early SSC differentiation. Moreover, we utilized biotin-labeled synthetic peptides to co-precipitate HM-binding proteins with low-input cell numbers. We further analyzed the essential roles of proteins, such as GTF2E2 and SUPT5H, in SSC differentiation. Together, our work provided necessary data resources for the subsequent study of meiosis in SSCs.

## 2. Results

### 2.1. Induction of SSC Differentiation In Vitro

**Figure 1:**
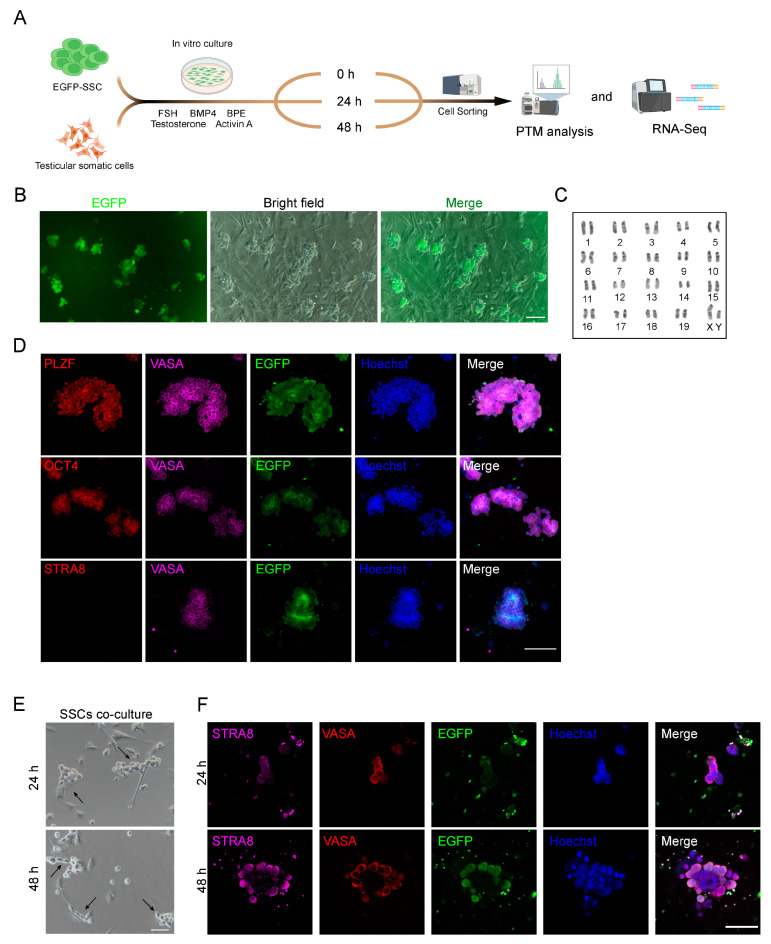
SSC culture and in vitro differentiation. (**A**) A schematic diagram for SSC differentiation in vitro. (**B**) In vitro fluorescence imaging of SSC expressing green fluorescence. Scale bar = 100 μm. (**C**) Karyotype analysis of EGFP-SSC. (**D**) Immunofluorescence staining of SSC stage-specific markers (VASA, OCT4, PLZF) and staining of spermatogonia differentiation marker STRA8. Scale bar = 100 μm. (**E**) Representative microscopic views of different stages of SSC differentiation in vitro. Scale bar = 50 μm. Arrowheads showed differentiated SSCs. (**F**) Immunofluorescence staining of spermatogonia differentiation marker STRA8 after 24 h and 48 h differentiation. Scale bar = 100 μm.

Our group previously employed a 2D culture system composed of primordial germ cell-like cells (PGCLC) and neonatal testicular somatic cells to enable germ cells to complete meiosis in vitro to obtain spermatid-like cells [3]. Subsequently, using this system, we co-cultured EGFP-SSCs with neonatal testicular somatic cells to simulate the in vitro differentiation of SSCs (Figure 1A). Prior to induction, we also verified the germ cell-specific properties of EGFP-SSCs (Figure 1B). Based on our karyotype analysis, EGFP-SSC contained 20 pairs of chromosomes, which were commensurate with the chromosomal features of normal male murine diploid cells (Figure 1C). Using immunofluorescence staining with SSC-specific markers, we subsequently revealed that the SSC cell line expressed elevated levels of the SSC stage-specific marker PLZF and the stem cell-specific marker OCT4. STRA8 was not stained during the initial SSC differentiation process (Figure 1D). Following 24 h of co-culture, SSCs migrated to somatic testicular cells. The testicular somatic cells surrounded and aggregated with SSCs to form new cellular clones after 48 h of differentiation. This indicated that the SSCs exhibited typical features of early SSC differentiation (Figure 1E). Following 24 h of co-culture, the SSC differentiation marker STRA8 was weakly expressed among differentiated cells, as evidenced by immunofluorescence staining. The STRA8 expression became considerably more prominent following 48 h of co-culture, indicating that the SSCs, at this point, underwent extensive differentiation (Figure 1F). In summary, we successfully established the SSC in vitro differentiation system.

### 2.2. RNA-seq Analysis of SSCs Differentiation In Vitro

**Figure 2:**
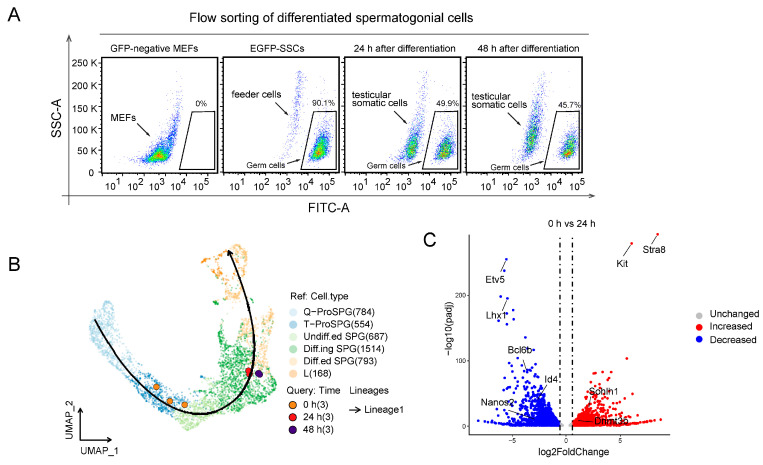

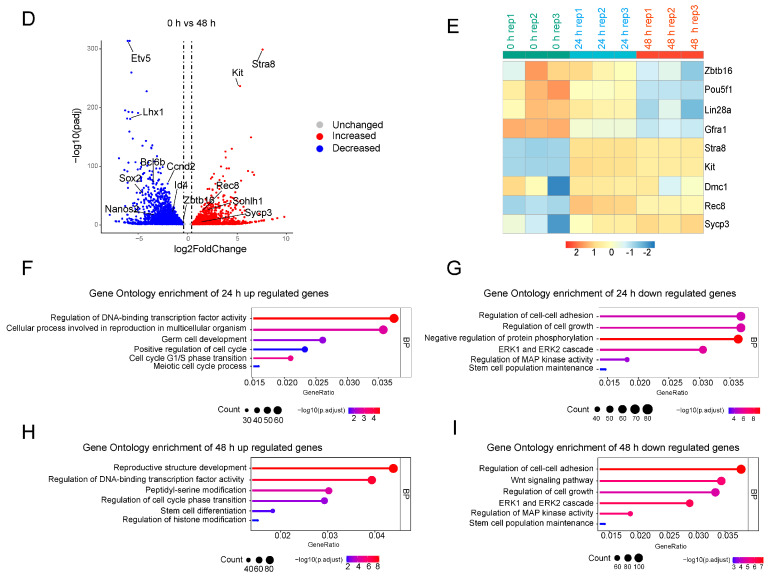
RNA-seq analysis of SSCs differentiation in vitro. (**A**) Flow sorting of differentiated spermatogonial cells and GFP-negative mouse embryonic fibroblast cells were used as control. (**B**) Comparison of RNA-seq data at three stages of differentiation in vitro with a reported in vivo single-cell RNA-seq data, the curve represents the differentiation trajectory. Q-ProSPG represents quiescent prospermatogonia, T-ProSPG represents transitional prospermatogonia, Undiff.ed SPG represents undifferentiated spermatogonia, Diff.ing SPG represents differentiating spermatogonia, Diff.ed SPG represents differentiated spermatogonia. (**C**) Volcano plot revealed genes that were up or down-regulated at 24 h of differentiation, with gene expression at 0 h as a control. (Foldchange ≤1.3 or >1.3, adjusted *p* valve < 0.05). (**D**) Volcano plot revealed differentially expressed genes at 48 h of differentiation, with gene expression at 0 h as a control. (Foldchange ≤1.3 or >1.3, adjusted *p* valve < 0.05). (**E**) Heat map analysis of spermatogenic cell-specific markers. (**F**,**G**) Gene Ontology enrichment analysis of up or down-regulated genes at 24 h. (**H**,**I**) Gene Ontology enrichment analysis of up or down-regulated genes at 48 h.

We next sorted SSCs at different stages of culture using flow cytometry for RNA-seq analysis (Figure 2A). To determine the stage of SSC differentiation, we first compared our RNA-seq data with reported single-cell sequencing data [8,10]. This revealed that the EGFP-SSCs were consistent with the transitional prospermatogonia stage (T-Pro SPG). Following a 24 h differentiation, they were at the differentiating spermatogonia stage (Diff.ing SPG). Lastly, after 48 h of co-culture, the EGFP-positive cells were closer to differentiated spermatogonia (Diff.ed SPG). To better illustrate the conclusion, we used two datasets from published works for comparison [9,11]. We found the SSCs differentiated for 48 h were in the differentiating spermatogonial stage, specifically in S-phase Type B spermatogonia, while 0-h SSCs were in the Type A spermatogonia stage (Figure S2A,B). These patterns were also consistent with the spermatogonia differentiation trajectory in vivo (Figure 2B). Based on the RNA-seq results, we obtained a total of 4647 differentially expressed genes between 0 h and 24 h, including 2017 upregulated genes and 2630 down-regulated genes, and also 5917 differentially expressed genes between 0 h and 48 h, including 2527 upregulated genes and 3390 down-regulated genes (Table S4). The SSC differentiation markers Stra8 and Kit were dramatically upregulated after 24 and 48 h of co-culture in vitro. However, the expressions of prospermatogonial stem cell-specific markers *Etv5*, *Lhx1*, *Bcl6b* [12], and stem cell-specific marker *Oct4*, *Nanos2* were significantly downregulated, relative to SSCs (Figure 2C,D). The clustering heat map also revealed that the levels of SSC-specific markers *Zbtb16*, *Gfra1*, and *Lin28a* decreased significantly from 0 to 48 h, whereas the contents of the meiotic stage biomarkers, namely, *Rec8*, *Dmc1*, and *Sycp3* continued to rise (Figure 2D,E). Likewise, using GO enrichment analysis, we revealed that the functional terms of early SSC differentiation, including stem cell differentiation and germ cell development, were significantly upregulated between 24 and 48 h, indicating that our co-culture system initiated the SSC differentiation process (Figure 2F,H). Moreover, we noticed that stem cell proliferation-related GO, such as stem cell population maintenance, regulation of MAP kinase activity [13], and regulation of cell growth, were markedly downregulated during this process. Together, these results indicated that SSCs were induced to differentiate in vitro, as was evidenced by the meiosis-induced alterations in gene expression and morphology.

### 2.3. Dynamic Landscape of Histone H3 Modifications during SSC Differentiation

**Figure 3:**
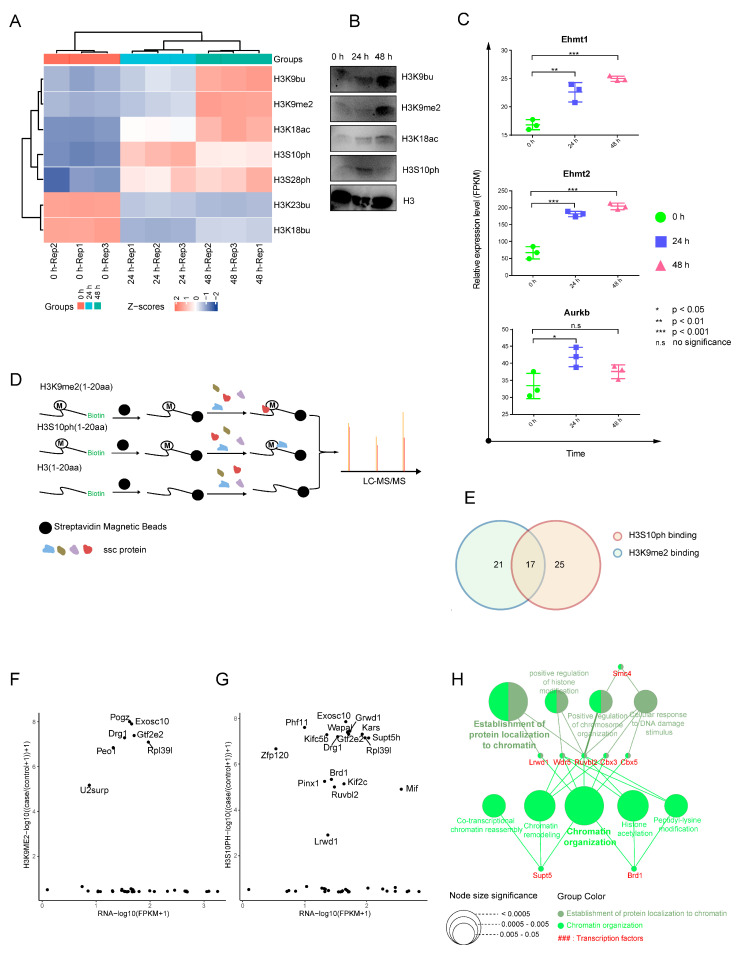
Histone modification profiling and enrichment of modification interacting proteins during SSC differentiation. (**A**) Heatmap of differentially expressed histone modifications at different stages of SSC differentiation. (**B**) Differentially expressed modifications identified by western blot validation. (**C**) Relative expression levels of histone modification enzymes EHMT1, EHMT2, and AURKB according to RNA–seq. The *y*–axis is the FPKM value. (**D**) Schematic diagram of the process of enriching binding proteins with synthetic two histone modification peptides. (**E**) Venn diagram of H3K9me2 and H3S10ph binding proteins. (**F**,**G**) Co–analysis of protein expression and RNA expression level. (**H**) Gene ontology of 8 transcription factors pulled down by H3K9me2 and H3S10ph peptides.

To elucidate the specific HM alterations that occur during SSC differentiation, we collected cells from three differentiation stages for targeted HM identification. We identified 36 histone H3 modifications from all three stages of differentiation (Table S1), among which seven differentially expressed (DE) modifications were H3K9bu, H3K9me2, H3K18ac, H3S10ph, H3S28ph, H3K23bu, and H3K18bu (Figure 3A and Figure S2A). We selected four modifications (H3K9me2, H3K18ac, H3S10ph, and H3K9bu) for further validation. Based on our analysis, the expression patterns were consistent with the trend we observed using mass spectrometry and Western Blotting (Figure 3B). These results indicated that the histone H3 modification underwent drastic alterations during SSC differentiation.

The H3K9me2, H3K9bu, H3K18ac, H3S10ph, and H3S28ph expressions gradually increased from 0–48 h (Figure 3A). Hence, we speculated that these modifications might contribute to SSC differentiation. We also observed that the H3K23bu and H3K18bu expressions diminished between 0–48 h (Figure 3A), suggesting that these two HMs were likely associated with SSC self-renewal. We also revealed alterations in the mRNA levels of histone H3.1 PTMs enzymes, which coincided with the trends in HMs. For example, the H3S10ph expression was deficient at 0 h, then peaked at 24 h, before slightly decreasing at 48 h. Based on our RNA-seq data, the phosphorylation kinase AURKB of H3S10ph gradually increased from 24 h, which corroborated with the elevated H3S10ph profile at 24 h (Figure 3C). The H3K9me2 expression gradually increased from 0 to 48 h, and its modification writers, EHMT1 and EHMT2, were also enhanced during these times (Figure 3C). These results further demonstrated the reliability of our histone H3.1 PTM data. In conclusion, we screened the HM profile during early SSC differentiation using targeted proteomics and identified multiple PTMs involving histone H3, which may potentially contribute to SSC differentiation.

### 2.4. Synthetic Biotin–Labeled Peptides Enriched in Proteins That Interacted with HMs

To further explore the interacting proteins associated with HMs, we synthesized biotin-labeled peptides for a pull-down examination with low-input cell number (1 × 10^5^). Our synthesized peptide interacted with target proteins and underwent biotin-streptavidin-based enrichment in vitro using C-terminal biotin labeling (Figure 3D). As reported previously, there is a “binary switch” relationship between H3K9me2 and H3S10ph, whereby H3S10ph can neutralize the inhibitory effect of H3K9me2, thereby enabling RNA polymerase II to extend to these regions for transcription [14]. Thus, we selected H3K9me2 and H3S10ph for subsequent experiments. We identified 38 H3K9me2-binding proteins, 42 H3S10ph-binding proteins, and 17 proteins that bind to both modifications (Figure 3E and Table S2) at the 48 h of SSC differentiation. We further analyzed the RNA expressions of these proteins and correlated that with corresponding protein expressions. Our analysis revealed that 63 of these proteins could be divided into two classes, one class that primarily concentrated in the baseline portion of the X-axis, suggesting that these proteins exhibited scarce expression regardless of mRNA levels, and another class was mainly gathered in the upper portion of the chart due to the relatively high expression of these proteins, indicating that these proteins may serve critical functions during SSC differentiation (Figure 3F,G). It is well established that HMs regulate gene transcription [15], and this effect is typically transmitted through transcription factors (TFs). We, therefore, speculated whether TFs were present among the modification-pull-down proteins. We revealed that, out of a total of 63 pull-down proteins, 8 were TFs, according to the CISTROME Database [16]. Based on our GO analysis, the biological processes associated with these eight TFs were mainly focused on chromatin organization, positive regulation of HM, and co-transcriptional chromatin reassembly (Figure 3H and Table S3).

**Figure 4:**
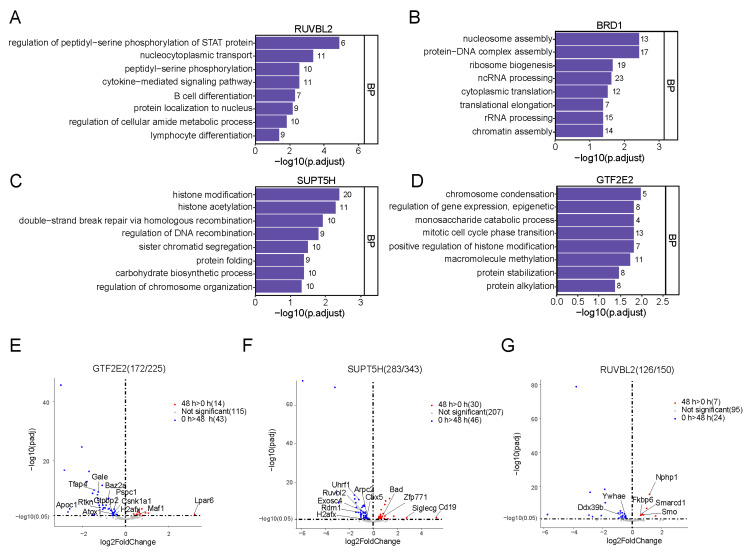

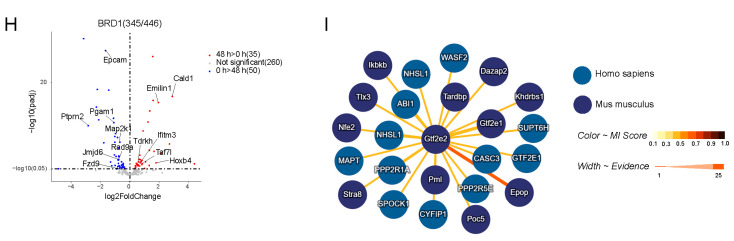
Gene function analysis of target genes regulated by four transcription factors. (**A**–**D**) GO enrichment for target genes regulated by four transcription factors through CISTROME database. (**E**–**H**) The expression level of target genes identified in our RNA-seq data. (**I**) Protein interaction prediction through IntAct Molecular Interaction database.

Among these eight TFs, four TFs exhibited relatively high protein levels. Hence, we selected these four TFs for further analysis. We first analyzed the GO of the target genes (downloaded from the CISTROME Database) associated with these four TFs. Based on the GO terms analysis, enrichments were mainly in protein modification (HM, histone acetylation, peptidyl-serine phosphorylation), cell differentiation (B cell differentiation, lymphocyte differentiation), cell cycle phase transition, and regulation of gene expression (Figure 4A–D). These results demonstrated that the transition process from prospermatogonia to differentiated spermatogonia essentially utilized HMs, namely H3K9me2 and H3S10ph. Moreover, their roles may be transmitted using TFs, such as GTF2E2, SUPT5H, BRD1, and RUVBL2. To elucidate whether the target genes associated with these four TFs also demonstrated significant alterations in our RNA-sequencing data, we mapped these target genes to our RNA-sequencing data. Red and blue dots represented highly- and scarcely-expressed genes at 48 h, respectively (Figure 4E–H). We also revealed that the genes with relatively high expression at 0 h were primarily related to cell growth and proliferation, particularly cancer cell proliferation, such as *Tfap4* [17], *Rdm1* [18], *Ddx39b* [19], *Pgam1* [20], and so on. Some of the highly expressed genes at 48 *h* were related to growth inhibition and differentiation, such as *Maf1* [21], *Zfp771* [22], *Smarcd1* [23], *Hoxb4* [24], and so on (Figure 4E). Lastly, we also revealed that the TF GTF2E2, which was pulled down by both H3K9me2 and H3S10ph, interacted with *Stra8* [25], and *Stra8* served essential modulatory roles in the initiation of SSC differentiation (Figure 4I). Therefore, we hypothesized that these TFs, particularly GTF2E2, regulate SSC differentiation by transmitting HM functions.

## 3. Discussion

Epigenetic regulation events [26], including de novo DM and HM, are early modulators of spermatogenesis [27]. To better reveal the role of histone H3.1 PTM in SSC differentiation, we screened histone H3.1 PTMs at different time points and identified 7 DE modification sites.

Among the seven DE HM sites we identified, our research primarily focused on H3K9me2 and H3S10ph. It is reported that in mice, repressive H3K9me2 is strongly expressed at the spermatogonia and early spermatocyte stages. However, following pachytene spermatocytes, the H3K9me2 expression is gradually attenuated and eventually confined to the pericentromeric region of chromosomes [28]. Prior investigations revealed that the methyltransferase EHMT2-induced H3K9me2 silences L1 elements in the absence of functional piRNA pathways and L1 DM. Moreover, the H3K9me2, piRNA pathways, and L1 DM coordinately inhibit the retrotransposon activities in SSC, and functional *Ehmt2* knockout results in spermatogenesis failure in mice [29]. Based on our in vitro differentiation-based transcriptome analysis, cells that differentiated for 48 h corresponded to the early leptotene spermatocytes in vivo, and the H3K9me2 expression gradually increased from 0 to 48 h, suggesting that the H3K9me2 may serve a critical role in spermatogonia differentiation. Furthermore, we observed that in the RNA-sequencing data, the H3K9me2 methyltransferase EHMT2 expression increased from 0–48 h, which was consistent with the H3K9me2 profile.

H3S10ph was previously reported as critical for mitosis [30]. Its vital phosphorylase AURKB is essential for assembling the synaptonemal complex during meiosis initiation. AURKB and another Aurora kinase, AURKC, cooperate to ensure the smooth assembly of the lateral axis of the synaptonemal complex [31]. AURKB and AURKC knockouts produce abnormal spermatogenesis. In our biotin-labeled data, AURKB was highly expressed at 24 h, which corroborated sufficiently with the elevated H3S10ph expression at 24 h. However, in mammals, the role of H3S10ph in meiosis remains poorly determined. Previous investigations confirmed that H3S10ph is not expressed until the pachytene stage of first meiosis. Moreover, from the late pachytene stage, H3S10ph is expressed in the pericentromeric regions, for example, the H3K9me2 localization in early spermatocyte chromosomes. This pattern of simultaneous H3S10ph and H3K9me2 expressions is termed a binary switch hypothesis and suggests that the serine residue phosphorylation can influence the inhibitory methylation marks on adjacent lysine residues. Interestingly, based on our histone H3 modification data, the H3K9me2 expression was the highest at 48 h, while H3S10ph was more pronounced at 24 h.

We enriched the interacting proteins of H3K9me2 and H3S10ph in SSC after 48 h of differentiation. Our analysis identified 63 H3K9me2- and H3S10ph-associated proteins. We also identified eight TFs using a pull-down assay. Our GO analysis of target genes associated with the identified TE revealed that four TFs were primarily focused on HM, cell differentiation, cell cycle regulation, and transcription regulation. The TF GTF2E2 was also able to interact with *Stra8*, which may regulate SSC differentiation. Therefore, we speculate that these are the enriched binding proteins of H3.1 modification peptides and provide an essential resource for studying the epigenetic regulation of histone H3 in the early stage of SSC differentiation. In future investigations, the functional evaluations of these proteins, along with the identification of potential proteins that may affect SSCs differentiation, are critical and necessary.

## 4. Materials and Methods

### 4.1. SSC Culture and Differentiation

EGFP-SSC was previously established by our team. EGFP-SSCs were maintained on mitomycin C (Sigma-Aldrich, Merck, Darmstadt, Germany)-exposed mouse embryonic fibroblasts in a 37 °C incubator with 5% carbon dioxide. The SSC culture medium was StemPro-34 SFM (StemPro™, Thermo Fisher Scientific, Waltham, MA, USA) with StemPro34 supplement (StemPro™, Thermo Fisher Scientific, Waltham, MA, USA), along with 25mg/mL insulin, 100 mg/mL transferrin, 60 mM putrescine, 30 nM sodium selenite, 6 mg/mL D-(L)-glucose, 30 mg/mL pyruvic acid, 1 mL/mL DL-lactic acid (Sigma-Aldrich, Merck, Darmstadt, Germany), 5 mg/mL bovine albumin (Sigma-Aldrich A3803, Merck, Darmstadt, Germany), 2mM L-glutamine, 5 × 10^−5^ M 2-mercaptoethanol, minimal essential medium vitamin solution (Invitrogen), MEM nonessential amino acid solution (Gibco™, Thermo Fisher Scientific, Waltham, MA, USA), 10^−4^ M ascorbic acid, 10 mg/mL D-biotin, 30 ng/mL β-estradiol, 60 ng/mL progesterone (Sigma-Aldrich, Merck, Darmstadt, Germany), 20 ng/mL mouse epidermal growth factor (R&D, Bio-techne, Minneapolis, MI, USA), 10 ng/mL human basic fibroblast growth factor (R&D, Bio-techne, Minneapolis, MI, USA), 10 ng/mL recombinant rat glial cell line-derived neurotrophic factor (GDNF) (R&D, Bio-techne, Minneapolis, MI, USA), and 1% FBS (Gibco™, 16141, Thermo Fisher Scientific, Waltham, MA, USA).

For the SSCs co-culture, 3–7 day postpartum (dpp) busulfan-exposed mouse testes [32] were extracted, followed by a two-step enzymatic digestion, as reported earlier [33]. Cells underwent filtration through a 40 μm cell strainer, then were centrifuged for collection. SSCs were then combined with busulfan-exposed mouse testicular somatic cells at a 1:1 ratio prior to culture in α-MEM (Gibco™, Thermo Fisher Scientific, Waltham, MA, USA) containing 10% KSR (KnockOut™, Thermo Fisher Scientific, Waltham, MA, USA), BMP-4/7 (20 ng/mL each, R&D, Bio-techne, Minneapolis, MI, USA), retinoic acid (10–6 M, Sigma-Aldrich, Merck, Darmstadt, Germany), activin A (100 ng/mL, R&D, Bio-techne, Minneapolis, MI, USA), testosterone (10 mM, Acros Organics, Fisher scientific, Waltham, MA, USA), FSH (200 ng/mL, Sigma-Aldrich, Merck, Darmstadt, Germany), and BPE (50 mg/mL, Corning Life Sciences, 354123, Minneapolis, MI, USA).

### 4.2. Immunostaining

Cells underwent a 15 min fixation in 4% paraformaldehyde (PFA) at room temperature (RT), followed by a 20 min blocking in 0.3% Triton X-100/2% BSA in PBS, and subsequent overnight (ON) 4 °C incubation in primary antibodies against OCT4 (1:200,P0056, Merck, Germany), PLZF (1:200, ab305064, Abcam), GFP (1:200 Millipore, Merck, Darmstadt, Germany), DDX4 (1:500 Abcam, ab27591, Minneapolis, MI, USA), and STRA8 (1:200 Abcam, ab49602, Minneapolis, MI, USA). The samples were then thrice PBS-rinsed and then exposed for 2 h to fluorescein isothiocyanate (FITC)-labeled secondary antibodies, Alexa Fluor 555, and Alexa Fluor 647 (Thermo Fisher Scientific, Waltham, MA, USA). The DNA underwent a 10 min counterstaining in 10 mg/mL Hoechst 33,342 (Thermo Fisher Scientific, Waltham, MA, USA), with three subsequent PBS rinses. Lastly, image capture was performed using a Zeiss LSM800 Meta inverted confocal microscope.

### 4.3. Karyotype

Cells were grown in a medium with 0.025% colchicine for 4 h, prior to a 30 min hypotonic exposure in 1% sodium citrate at RT. The cells were then fixed for 2 h in freshly made methanol/acetic acid (3:1) employing three fixative durations, followed by a 2 h rinse in ice water and subsequent evaluations. Chromosome visualization was performed via Giemsa staining. Image capture was performed via a Zeiss Axio Imager A1 microscope.

### 4.4. Flow Cytometry Analysis

Cells underwent dissociation in 0.25% trypsin, re-suspension in SSC culture medium, and filtration via a 40 μm cell strainer, before FACS analysis using the FACS Aria Fusion SOP (BD bioscience).

### 4.5. Western Blotting

1 μg of isolated proteins underwent separation on a 15% or 10% SDS-PAGE prior to transfer onto PVDF membranes (BIORAD, Shanghai, China 162-0177), which then underwent a 2h blocking in 5% skimmed milk, followed by an ON incubation at 4 °C with anti-Histone H3 (Proteintech, 17168-1-AP, Planegg-Martinsried, Germany) (1:10,000), anti-Phospho-Histone H3 (Ser10) (Cell Signaling Technology, 13576S, Damvers, MA, USA) (1:1000), Anti-Di-Methyl-Histone H3 (Lys9) (Abcam, ab1220, Cambridge, UK) (1:2000), and anti-acetyl-Histone H3 (Lys18) (PTM BIO, PTM-158) (1:2000). This was followed by four PBST-rinses, and subsequent 2 h secondary antibodies incubation (Goat Anti-Rabbit IgG H&L (HRP, Abcam, ab6721, 1:10,000, Cambridge, UK) and Goat Anti-Mouse IgG H&L (HRP, Abcam, ab6789, 1:10,000), Cambridge, UK) at RT. Again, the membranes were rinsed four times in PBST buffer before employing the High-sig ECL Western Blotting Substrate (Tanon) to identify protein bands.

### 4.6. Histone Acid Extraction

The extracted histone was identified via LC-MS/MS and western blot analyses, as reported by Shechter D.10 Briefly, SSCs underwent lysis in hypotonic lysis buffer (10 mM Tris–HCl pH 8.0, 1 mM KCl, 1.5 mM MgCl_2_, 1 mM DTT and cocktail) via a 30 min incubation on a rotator at 4 °C, before centrifugation at 10,000× *g* for 30 min at 4 °C. The pellet was further lysed in 0.4 NH2SO4 via sonication, followed by an ON rotator incubation and subsequent centrifugation at 16,000× *g* for 10 min at 4 °C. The supernatant from the acid extraction was then dialyzed for 2 h in dialysis tubing using low molecular weight cut-off against ddH_2_O.

### 4.7. Biotinylated H3K9me2 and H3S10ph Peptides Pull down Analysis

Biotinylated H3K9me2 and H3S10ph peptides were ON-incubated with Streptavidin-coated magnetic beads (Pierce) with rotation at 4 °C, followed by two rinses in RIPA lysis buffer and centrifugation. The cell pellets underwent lysis in RIPA lysis buffer (50mM Tris, 150 mM NaCl, 0.1%SDS, 0.5%SDC, 1%NP-40) prior to an ON incubation with beads while rotating at 4 °C, followed by five times rinsing in 25 mM Tris (PH 8.2), prior to digestion as previously described.

### 4.8. LC-MS/MS Analysis and Data Processing

To conduct LC-MS/MS analysis, peptides were introduced to 0.1% formic acid (FA) and assessed via an Orbitrap Fusion Lumos mass spectrometry (Thermo Fisher Scientific, Waltham, MA, USA) attached to the Easy-nLC 1200 (Thermo Fisher Scientific, Waltham, MA, USA). Solvent A had 0.1% FA in water, whereas solvent B had 80% ACN and 0.1% FA. Peptide separation was performed in an analytical column (75 μm × 25 cm, Acclaim PepMap RSLC C18 column, 2 μm, 100 Å; DIONEX, USA) with a 60 min linear gradient (3–5% B for 5 s, 5%–15% B for 23 min 55 s, 15–28% B for 21 min, 28–38% B for 7 min 30 s, 30–100% B for 5 s, and 100% B for 7 min 25 s) in the data-based acquisition mode. The Orbitrap Fusion Lumos was adjusted to 60K MS1 resolution with an AGC target of 4 × 10^5^ ions and maximum administration duration of 50 ms, with subsequent MS2 scans at 3K resolution, AGC target of 5 × 10^4^ ions, and maximum administration duration of 100 ms.

The default settings of the MaxQuant software (1.6.5.0) were used to process raw files, wherein mouse reference FASTA files were received from the Universal Protein Resource (UniProt) database in March 2021. Carbamidomethyl (C) was adjusted to fixed modifications, whereas, Oxidation (M), Acetyl (Protein N-term). Enzymatic specificity was defined as complete slicing via trypsin, and two maximum missed slicing locations were allowed.

### 4.9. Chemical Derivatization of Histones and Quantification by Parallel Reaction Monitoring

The extracted histone was derivatized via propionylation and digested with trypsin, as previously described. Each sample was combined with the isotope-labeled peptides and injected into an easy-nLC 1200 HPLC system (ThermoFisher Scientific, Waltham, MA, USA) via a 95 min gradient (3% to 5% buffer B for 5 s, 5% to 15% buffer B for 40min, 15% to 28% buffer B for 34 min 50 s, 28% to 38% buffer B for 12 min, 38% to 100% buffer B for 5 s, and 100% buffer B for 8 min). Analysis was carried out on an Orbitrap Fusion Lumos mass spectrometer as follows: higher-energy collisional at 30 eV. AGC target at 5.0 × 10^4^, scan range (*m*/*z*) at 150–2000.

The PRM data was processed using the Skyline Daily software. At least four transitions per precursor were used to quantify the targeted sample peptides. The quantitative levels of each modification of histone H3.1 were calculated as the ratio of the endogenous to heavy peptides, followed by normalization against the ratio of endogenous to the heavy peptide of the corresponding unmodified peptide.

### 4.10. RNA-seq and Data Analysis

Purified mRNA from the poly-T oligo-attached magnetic beads was employed for the sequencing library preparation by Novogene (Beijing, China). Upon library qualification, the different library pools based on the optimal concentration and target data quantity from the machine were sequenced via the Illumina NovaSeq 6000.

Three biological replicates were then analyzed for differential expression (DE) via the DESeq2 R package (1.20.0). DESeq2 employs a negative binomial distribution-based model to determine DE in gene expression datasets. The corresponding *p*-values were normalized based on Benjamini and Hochberg’s approach to minimize the false discovery frequency. Furthermore, padj ≤ 0.05 were deemed as significant DE.

We next employed Gene Ontology (GO) enrichment analysis of DE genes using the clusterProfiler R package (3.8.1), with gene length bias correction. GO terms with adjusted *p*-value < 0.05 were marked as significantly enriched by DE genes.

### 4.11. Differentiation Trajectory Analysis

We compared each sample of our RNA-seq data with each cell of the reference dataset, calculated the 30 nearest neighbors, and then performed dimension reduction through UMAP or TSNE to find the center point of these 30 nearest neighbors, which is each position of the time point on the graph. The R package used for data analysis is SCP (version 0.2.6), which can be obtained at the following address: https://github.com/zhanghao-njmu/SCP (accessed on 21 January 2023).

### 4.12. Data Availability

The RNA-seq data have been deposited to the GEO database under the accession number GSE222617. The raw mass spectrometry data were available via ProteomeXchange with the identifiers PXD03949 and PXD039496.

## Data Availability

Not applicable.

## Supplementary Materials

The following supporting information can be downloaded at: https://www.mdpi.com/article/10.3390/ijms24043314/s1.
