# Supplementary material for: Multi-Omics Approaches for Revealing the Epigenetic Regulation of Histone H3.1 during Spermatogonial Stem Cell Differentiation In Vitro
Source: Int J Mol Sci. 2023 Feb 7;24(4):3314. doi: 10.3390/ijms24043314 (PMC9958608; doi:10.3390/ijms24043314)
Supplement: Supplementary file 1 [file ijms-24-03314-s001.zip › Figure S2.pdf]

Figure S2

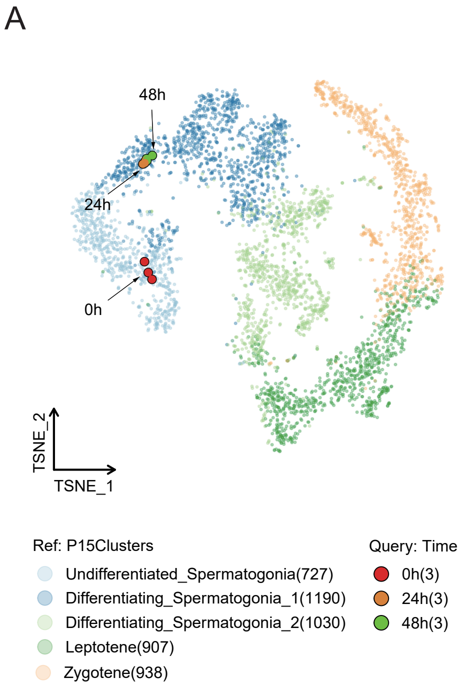
A
48h
24h
0h
TSNE_2
TSNE_1
Ref: P15Clusters
Undifferentiated_Spermatogonia(727)
Differentiating_Spermatogonia_1(1190)
Differentiating_Spermatogonia_2(1030)
Leptotene(907)
Zygotene(938)
Query: Time
0h(3)
24h(3)
48h(3)

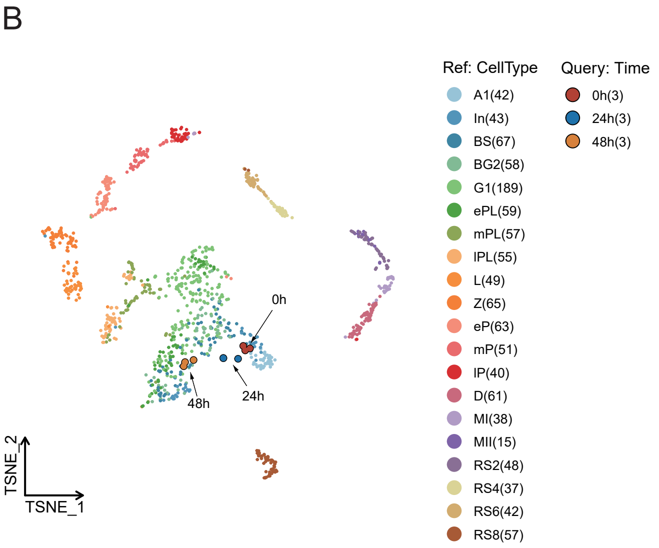
B
0h
48h
24h
TSNE_2
TSNE_1
Ref: CellType
A1(42)
In(43)
BS(67)
BG2(58)
G1(189)
ePL(59)
mPL(57)
lPL(55)
L(49)
Z(65)
eP(63)
mP(51)
lP(40)
D(61)
MI(38)
MII(15)
RS2(48)
RS4(37)
RS6(42)
RS8(57)
Query: Time
0h(3)
24h(3)
48h(3)
