# Supplementary material for: Multi-Omics Approaches for Revealing the Epigenetic Regulation of Histone H3.1 during Spermatogonial Stem Cell Differentiation In Vitro
Source: Int J Mol Sci. 2023 Feb 7;24(4):3314. doi: 10.3390/ijms24043314 (PMC9958608; doi:10.3390/ijms24043314)

Supplementary Figure

Multi-omics approaches for revealing the epigenetic regulation of histone H3.1 during spermatogonial stem cell differentiation in vitro

Figure S1: Extracted ion chromatograms of histone post-translational modification peptides by PRM

(A) Representative extracted ion chromatograms of histone post-translational modification peptides from 0h, 24h, and 48h differentiated SSCs. Heavy peptide with BSA is used as a negative control.

Figure S1

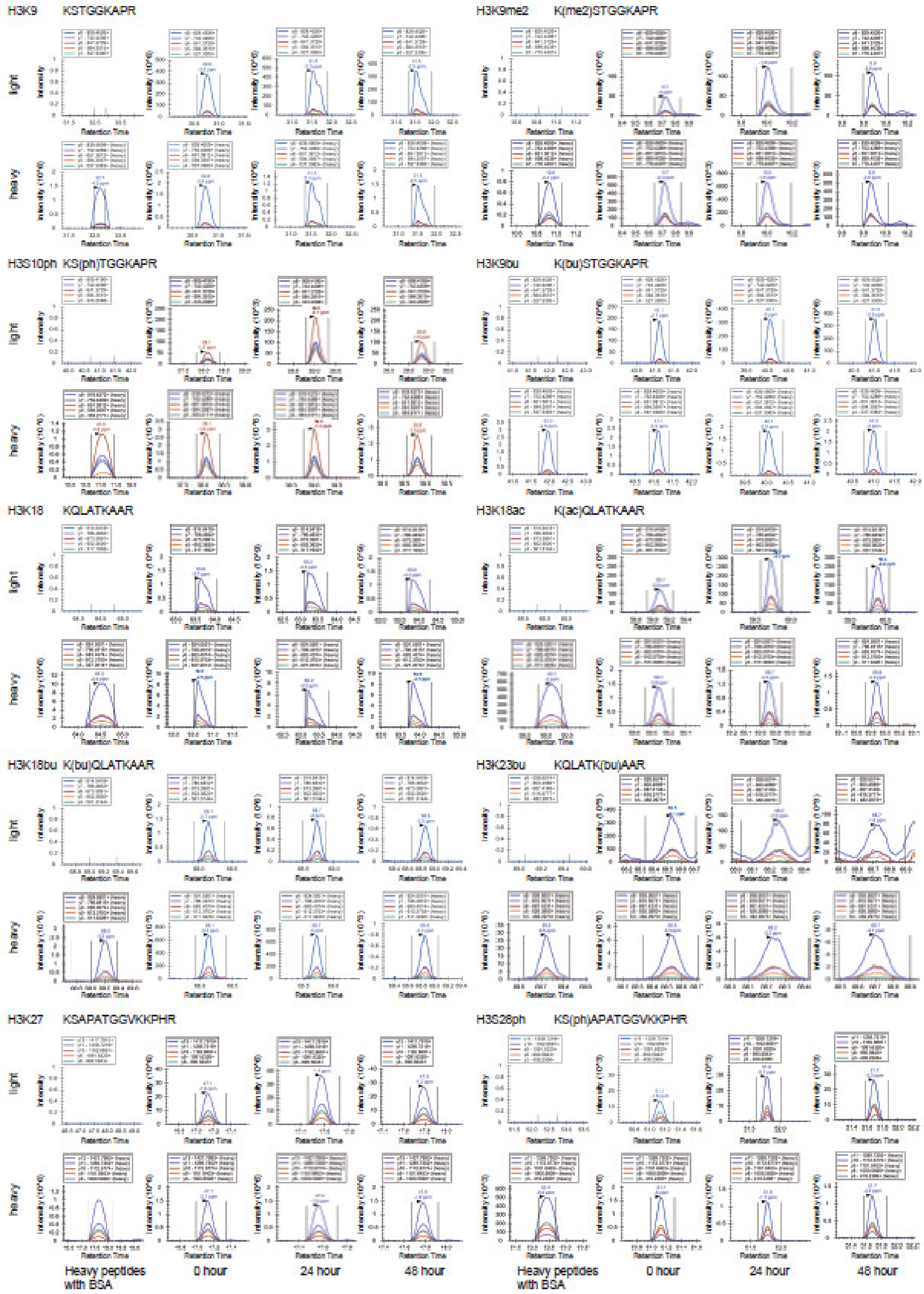

H3K9 KSTGGKAPR
H3K9me2 K(me2)STGGKAPR
H3S10ph KS(ph)TGGKAPR
H3K9bu K(bu)STGGKAPR
H3K18 KQLATKAAR
H3K18ac K(ac)QLATKAAR
H3K18bu K(bu)QLATKAAR
H3K23bu KQLATK(bu)AAR
H3K27 KSAPATGGVKKPHR
H3S28ph KS(ph)APATGGVKKPHR
light
heavy
Retention Time
Intensity
Heavy peptides with BSA
0 hour
24 hour
48 hour

Figure S2: Differentiation trajectory of SSCs compared with another two published datasets.

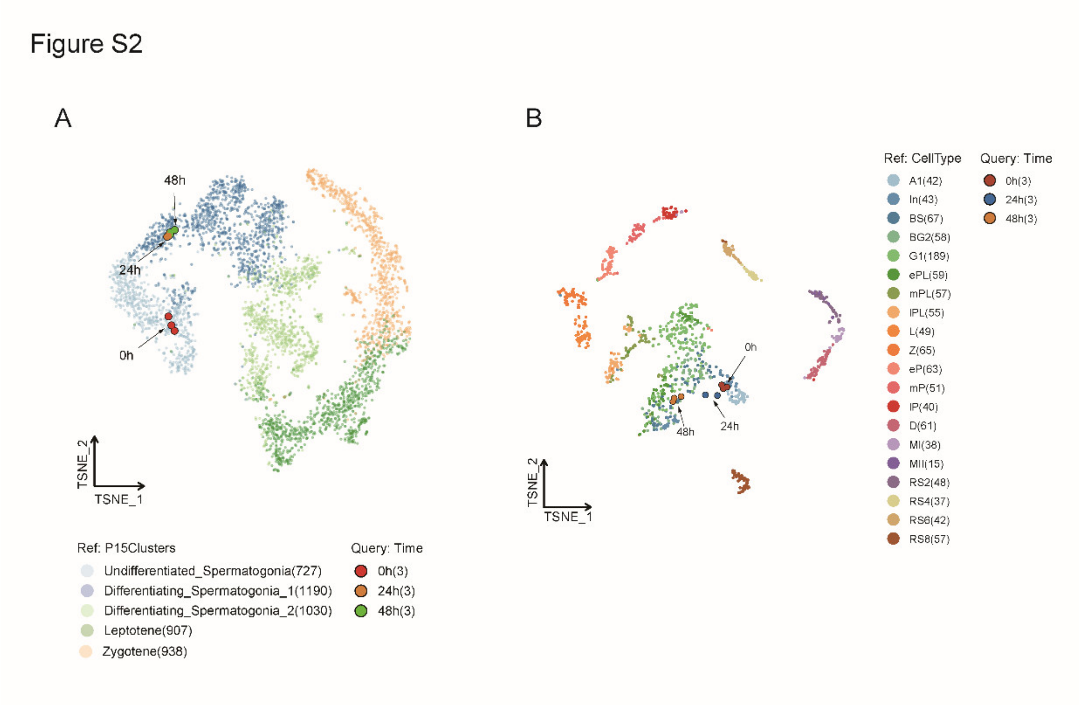

Supplement: Supplementary file 1 [file ijms-24-03314-s001.zip › Supplementary Figure.pdf]
