# Supplementary figures and images for: Multi-Omics Approaches for Revealing the Epigenetic Regulation of Histone H3.1 during Spermatogonial Stem Cell Differentiation In Vitro
Source: Int J Mol Sci. 2023 Feb 7;24(4):3314. doi: 10.3390/ijms24043314 (PMC9958608; doi:10.3390/ijms24043314)

Figure S1

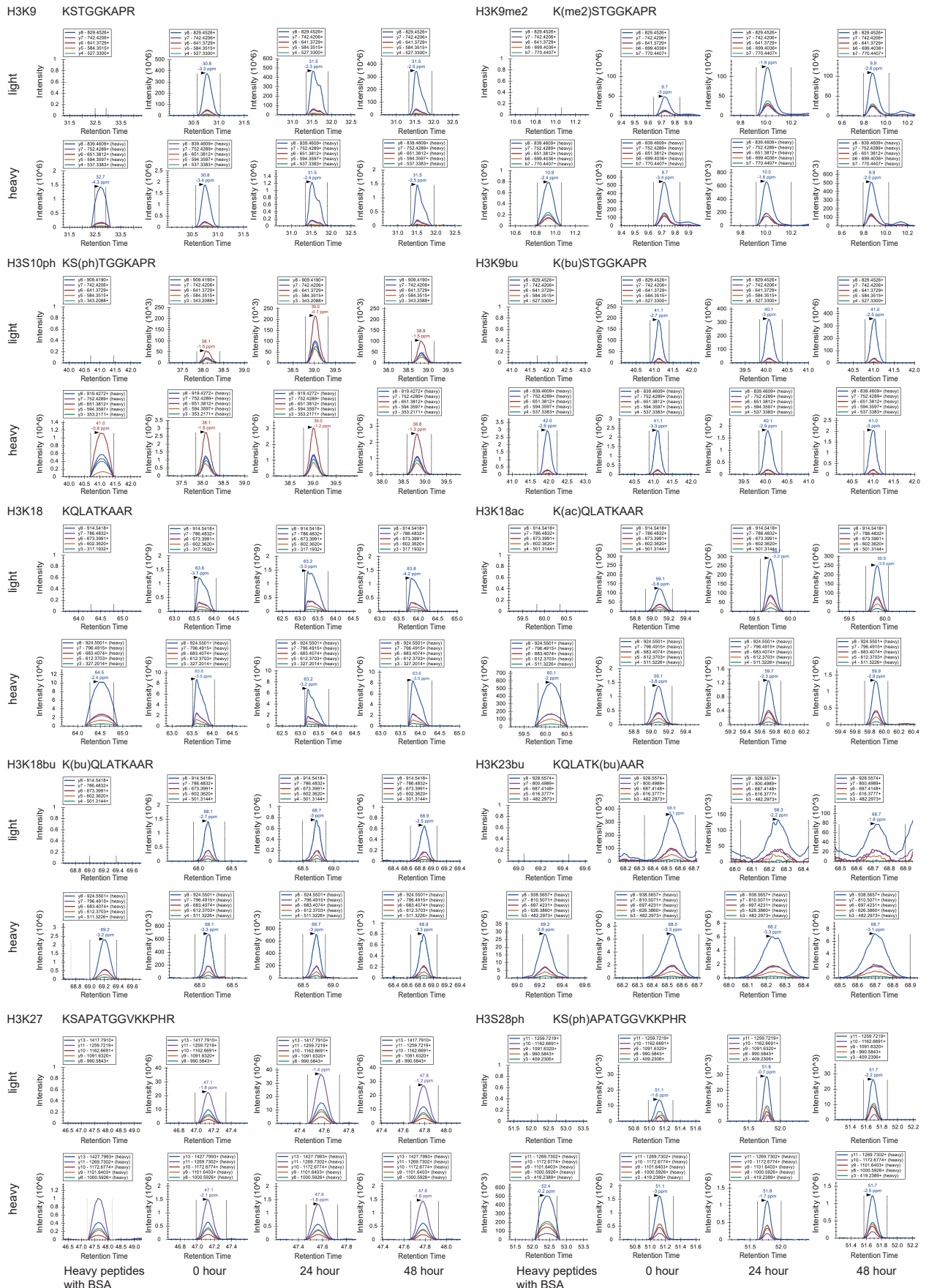

Supplement: Supplementary file 1 [file ijms-24-03314-s001.zip › Figure S1.pdf]
